# Spin-Polarized Transport and Spin Seebeck Effect in Triple Quantum Dots with Spin-Dependent Interdot Couplings

**DOI:** 10.1186/s11671-018-2773-1

**Published:** 2018-11-08

**Authors:** Li-Ming Liu, Feng Chi, Zhen-Guo Fu, Shu-Chao Yu, Hong-Wei Chen

**Affiliations:** 1 School of Electronic and Information Engineering, University of Electronic Science, and Technology of China, Zhongshan Institute, Zhongshan, 528400 China; 2 Institute of Applied Physics and Computational Mathematics, Huayuan Road 6 Haidian District, Beijing, 100088 China; 3 State Key Laboratory of Electronic Thin Films and Integrated Device, University of Electronic Science and Technology of China, Chengdu, 610054 China

**Keywords:** Spin Seebeck effect, Quantum dots, Spin-dependent interdot coupling, Pure spin thermopower

## Abstract

We study the spin-dependent electronic and thermoelectric transport through a structure composed of triple quantum dots (TQDs) coupled to two metallic leads in the presence of spin-dependent interdot couplings, which is reliable by applying a static magnetic field on the tunnel junctions between different dots. When the TQDs are serially connected, a 100 *%* spin-polarized conductance and thermopower emerge even for very small spin-polarization of the interdot coupling as the dots are weakly coupled to each other. Whereas if the TQDs are connected in a ring shape, the Fano antiresonance will result in sharp peaks in the conductance and thermopower. In the presence of spin-dependent interdot couplings, the peaks of the spin-up and spin-down thermopowers will shift to opposite directions in the dot level regime, resulting large either 100 *%* spin-polarized or pure spin thermopowers. The latter generally arises at low temperatures and is robust against the level detuning, the dot-lead coupling, and the system equilibrium temperature.

## Introduction

Along with the development of spintronics [1–3], spin caloritronics [4, 5] has been paid much attention during the last two decades. In spintronics, one of the most attractive issues is to control electron spin by electrical bias. Whereas in spin caloritronics, the spin control method is mainly the thermal bias, a temperature gradient applied between different ends of the system. It is regarded as a combination of spintronics and thermoelectricity. Of particular interest is the spin Seebeck effect (SSE) that generates pure spin current without the accompany of the charge counterpart, or spin bias characterized by the splitting of spin-up and spin-down chemical potentials. It opens a way of utilizing the excess heat generated in nanostructures to achieve lower-energy consumption and improved performance in thermal devices. Such kind of device is also effective in detecting the system temperature gradient with the help of carriers’ spin degree of freedom. Since 2008, some great experimental breakthroughs of the observation of SSE were continuously reported by K. Uchida et.al. in magnetic metals [6], ferromagnetic insulators [7, 8], and ferromagnetic metals [9]. It was subsequently studied in ferromagnetic semiconductors [10], nonmagnetic materials with a magnetic field [11], paramagnetic materials [12], antiferromagnetic materials [13], metal-ferromagnet insulator interface [14], and also topological insulators [15–17].

It was proved by Mahan and his coworker that a delta-like shape of the transmission function, which is common in low-dimensional systems, will remarkably enhance the efficiency of thermoelectric devices [18]. Since then, the zero-dimensional quantum dot (QD) [19, 20] in which the carries are confined in all three dimensions has been extensively studied to enhance the SSE coefficient (spin thermopower), which indicates the magnitude of generated spin bias under the condition of open circuit by the infinitely small thermal bias [4–6]. Especially, if there are more than one transmission paths in the system, the electrons will interfere with each other and may arise the interesting Dick [21, 22] or Fano [23, 24] effects characterized by sharp change of the transmission function and conductance. Therefore, much work has been devoted to the investigation of SSE in various ring-shape or multiple-path structures containing QDs [25–33]. The rich parameters in it, such as the tunable dot levels, Coulomb interaction, magnetic flux, spin-orbit interactions, asymmetry of the dot-lead couplings enable effective control of the quantum interference processes, resulting in giant spin thermopower whose magnitude can reach as high as or even higher than that of the charge one.

**Fig. 1:**
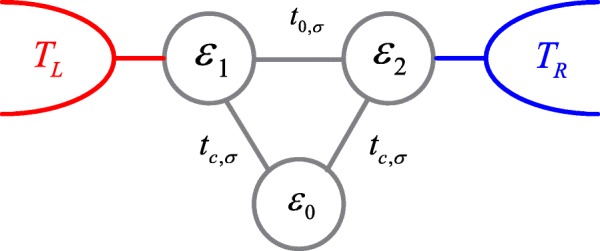
Schematic plot of the triple quantum dots system. By applying a static magnetic field on the tunnel barriers between the dots, the interdot couplings become spin-dependent

Triple QDs (TQDs) with various shapes have been prepared in experiments and theoretically studied which focus on the stability diagram, charge rectification, charge frustration, quantum interference effect, and coherent spin control [34–46]. Among them, the dots connected in a ring shape is more interesting due the existence of quantum interference effect [39–46]. As compared to the electron transport, the thermoelectric effect, especially SSE has seldom been studied in TQDs. In the present paper, we investigate the SSE in TQDs taking spin-dependent interdot couplings into consideration (see Fig. 1). By applying a static magnetic field on the tunnel junctions between QDs, the electron spin perform Larmor precession, and the interdot couplings become spin-dependent [47, 48]. Recently, it was also proposed that by utilizing oscillating magnetic fields and temporally controlled gate voltages, one can separate the electron wave functions of different spin component into different QDs, inducing spin-resolved transfer speed (coupling strength) [49, 50]. In some previous work, the effects of spin-dependent interdot coupling on the generation of spin current has already been investigated [51, 52]. Here, we show that it can shift the positions of the spin-up and spin-down thermopowers to opposite directions in dot level space by changing the Fano antiresonance states, resulting in 100 *%* spin-polarized or pure spin thermopowers whose magnitude can be as large as that of the charge one. Such an effect is quite different from the case of spin-independent interdot coupling [53, 54]. Interestingly, the obtained results can be fulfilled with very small spin-polarization of the interdot couplings.

## Model and Methods

The Hamiltonian of the TQDs shown in Fig. 1 connected to two leads may be modelled by the following Anderson Hamiltonian [25, 33, 51, 52],

1 $$ \begin{aligned} H=\!\!\sum\limits_{k\beta\sigma}\varepsilon_{k\beta}c_{k\beta\sigma}^{\dag}c_{k\beta\sigma}\!\,+\,\!\!\sum\limits_{i\sigma}\varepsilon_{i}d_{i\sigma}^{\dag}d_{i\sigma} \!\,+\,\!\!\sum\limits_{\sigma}\!(t_{0,\sigma}d_{1\sigma}^{\dag} d_{2\sigma}\!\,+\,t_{c,\sigma}d_{1\sigma}^{\dag} \!d_{0\sigma}\\ + t_{c,\sigma}d_{0\sigma}^{\dag} d_{2\sigma}\!\,+\,H.c)\,+\,\!\!\sum\limits_{k,\sigma}\left(V_{kL}c_{kL\sigma}^{\dag}d_{1\sigma}\!\,+\,\!V_{kR}c_{kR\sigma}^{\dag}d_{2\sigma}\!\,+\,\!H.c\right), \end{aligned}  $$

where $c_{k\beta \sigma }^{\dag } \left (c_{k\beta \sigma }\right)$ with *β*=*L*,*R* and $d_{i\sigma }^{\dag } \left (d_{i\sigma }\right)$ with *i*=0,1,2 are respectively the creation (annihilation) operators in lead- *β* and dot-*i* with spin *σ*. We assume that each dot includes a single energy level *ε*_*i*_ and neglects the Coulomb interaction between the electrons in the dots and the leads. QD-1 and QD-2 are coupled to each other by the interdot coupling *t*_0,*σ*_=*t*_0_(1+*σp*) and to the left and right leads by the dot-lead coupling *V*_*kL*_ and *V*_*kR*_, respectively. The QD-0 is connected to QD-1 and QD-2 with strength *t*_*c*,*σ*_=*t*_*c*_(1+*σp*), where *σ*=±1 for spin-up and spin-down electrons, respectively.

In the linear response regime, we can individually write the spin-dependent electric and heat currents under infinitely small potential difference *ΔV* and a temperature difference *ΔT* between the left and right leads as [25, 33]

2 $$\begin{array}{*{20}l} &&J_{e,\sigma}=-e^{2}K_{0,\sigma}\Delta V+\frac{e}{T}K_{1,\sigma}\Delta T, \end{array} $$

3 $$\begin{array}{*{20}l} &&J_{h,\sigma}=eK_{1,\sigma}\Delta V-\frac{1}{T}K_{2,\sigma}\Delta T, \end{array} $$

where *e* is the electron charge and *T* the system equilibrium temperature. The coefficients *K*_*n*,*σ*_ in the above equation are given by [25, 33]

4 $$\begin{array}{@{}rcl@{}} K_{n,\sigma}=\frac{1}{\hbar}\int (\varepsilon-\mu)^{n}[-\frac{\partial f(\varepsilon,\mu)}{\partial \varepsilon}]T_{\sigma}(\varepsilon)\frac{d\varepsilon}{2\pi}, \end{array} $$

where $\hbar $ is the reduced Planck’s constant, *μ* the leads’ chemical potential, *f*(*ε*,*μ*)=1/{1+exp[(*ε*−*μ*)/*k*_*B*_*T*]} the Fermi distribution function with Boltzmann constant *k*_*B*_.

In Eq. (4), the transmission coefficient *T*_*σ*_(*ε*) for each spin component can be obtained in terms of the retarded Green’s function as [25, 33] $T_{\sigma }(\varepsilon)=\Gamma _{L}\Gamma _{R} \left |G_{21,\sigma }^{r}(\varepsilon)\right |^{2}$, where $\Gamma _{L(R)}=2\pi \sum _{k}|V_{kL(R)}|^{2}\delta \left [\varepsilon -\varepsilon _{kL(R)}\right ]$ is the line-width function. Applying the equation of motion method, we can easily derive the analytical form of $G_{21,\sigma }^{r}(\varepsilon)$ as [55, 56]

5 $$ G_{21,\sigma}^{r}(\varepsilon)= \frac{\left(\varepsilon-\varepsilon_{0}\right)t_{0,\sigma}+t_{c,\sigma}^{2}}{\left(\varepsilon-\varepsilon_{0}\right)\left(\tilde{\varepsilon}_{1}\tilde{\varepsilon}_{2}-t_{0,\sigma}^{2}\right)-t_{c,\sigma}^{2}\left(\tilde{\varepsilon}_{1}+\tilde{\varepsilon}\right)-2t_{0,\sigma}t_{c,\sigma}^{2}},  $$

where $\tilde {\varepsilon }_{1(2)}=\varepsilon -\varepsilon _{1(2)}+i\Gamma _{L(R)}/2$. The transmission coefficient then is obtained as [55, 56]

6 $$\begin{array}{@{}rcl@{}} T_{\sigma}(\varepsilon)=\frac{\Gamma_{L}\Gamma_{R}[\left(\varepsilon-\varepsilon_{0}\right)t_{0,\sigma} +t_{c,\sigma}^{2}]^{2}}{\left|\left(\varepsilon-\varepsilon_{0}\right)\left(\tilde{\varepsilon}_{1}\tilde{\varepsilon}_{2}-t_{0, \sigma}^{2}\right)-t_{c,\sigma}^{2}\left(\tilde{\varepsilon}_{1}+\tilde{\varepsilon}\right)-2t_{0,\sigma}t_{c,\sigma}^{2}\right|^{2}}, \end{array} $$

The thermopower (Seebeck coefficient) of each spin component *S*_*σ*_ is calculated under the condition of vanishing charge current *J*_*e*_=*J*_*e*,*↑*_+*J*_*e*,*↓*_=0, and is given by [25, 33] *S*_*σ*_=−*K*_1,*σ*_/(*eTK*_0,*σ*_), and the charge (spin) thermopower is given by *S*_*c*(*s*)_=*S*_*↑*_+(−)*S*_*↓*_.

## Results and Discussions

**Fig. 2:**
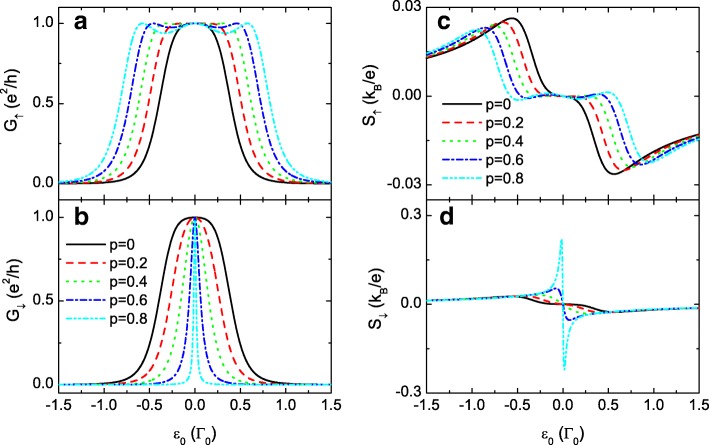
Conductance and thermopower for *t*_0_=0. Spin-polarized conductance *G*_*σ*_ in **a** and **b**, and thermopower *S*_*σ*_ in **c** and **d** as functions of the dot level *ε*_0_ for fixed *t*_0_=0 and different values of the spin-polarization of the interdot couplings. The other parameters are level detuning *Δ*=0, temperature *T*=0.001, and *t*_*c*_=0.3

In the following numerical calculations, we choose the line-width function *Γ*_*L*_=*Γ*_*R*_=*Γ*_0_=1 as the energy unit and fix *μ*=0 as the energy zero point. The constants of *e*, *k*_*B*_, and *h* are all set to be 1. Figure 2 shows the spin-dependent conductance *G*_*σ*_ and thermopower *S*_*σ*_ as functions of the dot level *ε*_0_=*ε*_1_=*ε*_2_ for *t*_0_=0, i.e., the TQDs are connected in series. When the interdot couplings are independent of spin (*p*=0), the spin-up and spin-down conductances in (a) and (b) are the same and develop a peak centered at *ε*_0_=0 (black solid lines).

In the presence of the spin-dependent interdot coupling *p*≠0, the single peak of the spin-up conductance *G*_*↑*_ in Fig. 2a evolves to a triple peak configuration with unchanged maximum peak value because of the enhanced spin-up interdot coupling *t*_*c*,*↑*_. Whereas *G*_*↓*_ remains the single-peak pattern with reduced peak width because of the smaller *t*_*c*,*↓*_. For *t*_0,*σ*_=0 and identical QDs levels (*ε*_1_=*ε*_2_=*ε*_0_), the transmission coefficient in Eq. (6) reduces to

7 $$\begin{array}{@{}rcl@{}} T_{\sigma}(\varepsilon)=\frac{\Gamma_{0}^{2}t_{c,\sigma}^{4}}{\left\{\left(\varepsilon-\varepsilon_{0}\right)\left[\left(\varepsilon-\varepsilon_{0}\right)^{2}-\Gamma_{0}^{2}/4\right]-2t_{0,\sigma}^{2}\right\}^{2}+\Gamma_{0}^{2}t_{c,\sigma}^{4}}. \end{array} $$

There are three resonances in the transmission function located respectively at *ε*=*ε*_0_ and $\varepsilon =\varepsilon _{0}\pm \sqrt {2t_{c,\sigma }^{2}+\Gamma _{0}^{2}/4}$. Under the condition of low temperature, three resonant peaks emerge in the conductance at *ε*_0_=*μ* and $\varepsilon _{0}=\mu \pm \sqrt {2t_{c,\sigma }^{2}+\Gamma _{0}^{2}/4}$, respectively. For the case of weak interdot coupling, the three peaks merge into a single-peak configuration as shown by the black lines in Fig. 2a and. With increasing interdot spin-polarization *p*, the value of *t*_*c*,*↑*_=*t*_*c*_(1+*p*) increases and the three peaks in the spin-up conductance are separated in energy space as shown in Fig. 2a. Meanwhile, the magnitude of *t*_*c*,*↓*_ becomes smaller and *G*_*↓*_ in Fig. 2b remains a single-peak pattern accordingly. From Eq. (6) one can also see that the peak width is reduced by decreasing *t*_*c*,*↓*_.

When *p*=0, the thermopowers of each spin component in Fig. 2c and d are identical and antisymmetric with respective to the electron-hole symmetry point (*ε*_0_=0), which is consistent with previous works [33, 57]. Due to the existence of temperature gradient that generates the thermoelectric effect, the temperature of the left lead is higher than that of the right one, and there are more electrons above the chemical potential *μ* in the left lead. Correspondingly, there are more holes below *μ*. When the energy levels of QDs are below (above) *μ*, the main carriers are holes (electrons) and then the thermopower is positive (negative) [57]. The thermopowers change their signs at *ε*_0_=0 due to the compensation of electrons and holes. With increasing *p*, the peak width of the spin-up thermopower *S*_*↑*_ is enlarged with reduced peak value. Whereas that of the spin-down is narrowed. Interestingly, the peak value of *S*_*↓*_ is obviously enhanced by increasing *p*. For the case of large interdot spin polarization, such as *p*=0.8, the peak value of *S*_*↓*_ is about ten times of *S*_*↑*_ with nearly unchanged value of the spin-dependent conductance *G*_*σ*_. This can be explained as follows. For positive *p*, the interdot tunneling rate *t*_*c*,*↑*_>*t*_*c*,*↓*_ and the spin-up electrons (or holes) will pass through the QDs quicker than the spin-down ones. Correspondingly, there are more spin-down electrons (holes) being blockaded at the left (right) leads as compared to the spin-up ones, resulting in larger spin-down voltage in response of the temperature gradient.

**Fig. 3:**
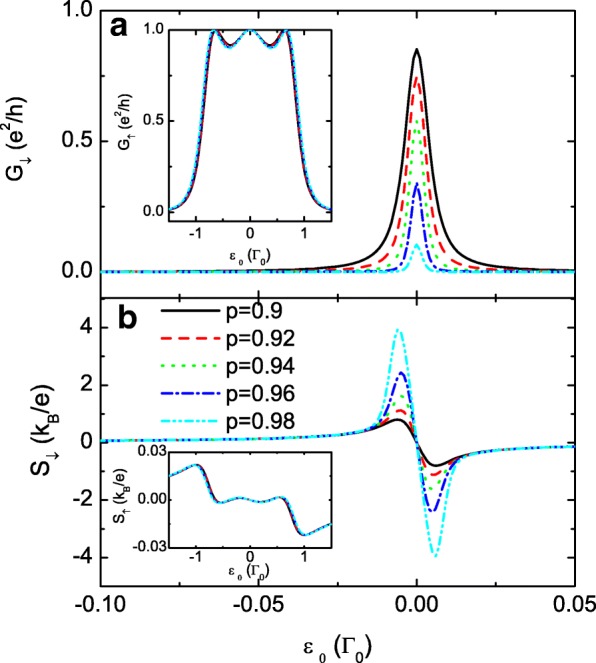
Spin-down conductance and the thermopower. The spin-down conductance *G*_*↓*_ in **a** and the thermopower *S*_*↓*_ in **b** for the case of large interdot coupling 1>*p*≥0.9. The inset in **a** is for *G*_*↑*_ in a large dot level regime, and the inset in **b** denotes the spin-up thermopower in comparison with the spin-down one. The other parameters are as in Fig. 2

**Fig. 4:**
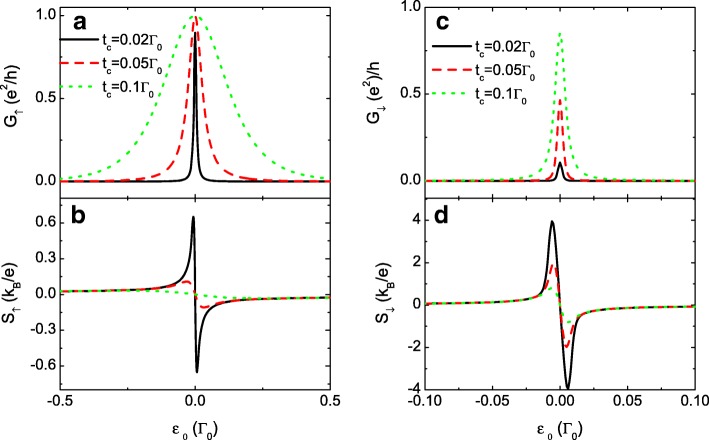
Conductance and the thermopower for different *t*_*c*_. Spin-polarized conductance *G*_*σ*_ in **a** and **c**, and the thermopower *S*_*σ*_ in **b** and **d** as functions of the dot level *ε*_0_ for *p*=0.7 and different values of *t*_*c*_. The other parameters are as in Fig. 2

To further enlarge the difference between *S*_*↓*_ and *S*_*↑*_, we present the results of extremely large *p* in Fig. 3. We find that the spin-up conductance *G*_*↑*_ and thermopower *S*_*↓*_ are less influenced by the variation of *p*, which is shown by the insets in Fig. 3a and b for comparison. With increasing *p*, the spin-down carriers become even harder to transport through the QDs and will be accumulated on the leads. Accordingly, the value of *G*_*↓*_ is monotonously suppressed, but the peak value of *S*_*↓*_ is remarkably enlarged, suggesting an effective means for generating a fully spin-polarized thermopower by the spin-dependent interdot coupling. This result may also be promising in detecting the temperature gradient in the system by SSE technique. Now that weak interdot coupling enhances the thermopower value, we then choose smaller *t*_*c*_ with fixed *p*=0.7 in Fig. 4. In this case, the three resonant peaks in both the spin-up and spin-down conductances are emerged into one. The peak width of the conductance is broadened by increasing *t*_*c*_ which is in agreement with previous results. Fig. 4b and d shows that the magnitude of both *S*_*↑*_ and *S*_*↓*_ is enhanced by decreasing *t*_*c*_. The maxima of the spin-down thermopower can also reach about 4 *k*_*B*_/*e* for *t*_*c*_=0.02*Γ*_0_. In experiments, the interdot couplings are adjustable by the gate voltage or the thickness of the tunnel barrier. Therefore, it may be more feasible to enhance the thermopower by changing *t*_*c*_ with a fixed spin-polarization *p*, as the magnetic field usually is more difficult to be controlled as compared to the electric field. In fact, large thermopower may be obtained with very small *p* under some conditions, as shown in the following.

**Fig. 5:**
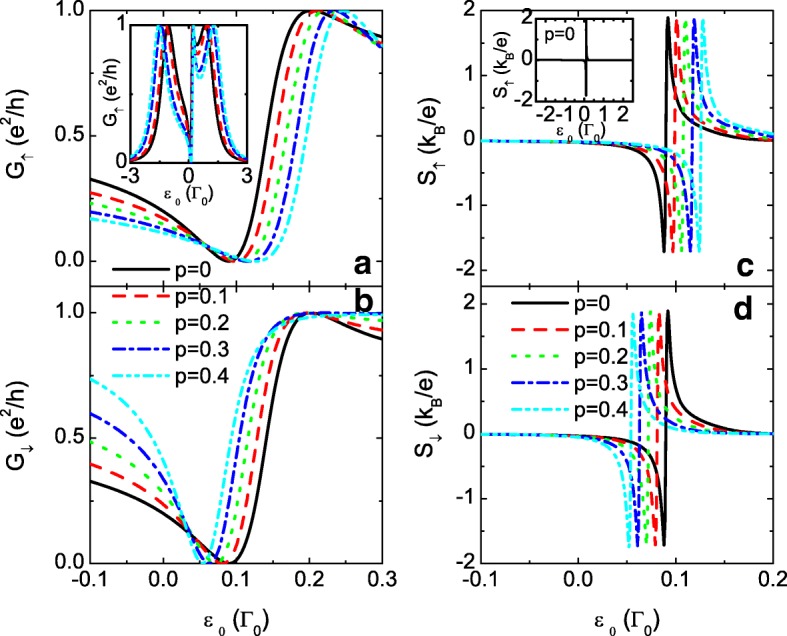
Conductance and the thermopower for *t*_0_=1. Spin-polarized conductance *G*_*σ*_ in **a** and **b**, and the thermopower *S*_*σ*_ in **c** and **d** as functions of the dot level *ε*_0_ for *t*_0_=1, *t*_*c*_=0.3 and different values of the spin polarization of the interdot couplings *p*. The insets in **a**and **c** are the conductance and the thermopower in a large dot level regime respectively. The other parameters are as in Fig. 2

If the QDs are connected in a ring shape, the arisen Fano effect will drastically change the properties of the conductance [46] and the thermopower. Particularly, giant thermopwer occurs around the Fano antiresonance state where the transmission function approaches to zero *T*_*σ*_(*ε*)=0 due to the complete reflection [25–33]. Replacing the electron energy *ε* by the chemical potential *μ* in Eq. (5), one can find the only antiresonance state is located at

8 $$\begin{array}{@{}rcl@{}} \varepsilon_{0}=\mu+t_{c,\sigma}^{2}/t_{0,\sigma}, \end{array} $$

which is determined solely by the interdot couplings and independent of the other parameters, such as the dot levels *ε*_1_, *ε*_2_, temperature *T* or the dot-lead hybrid matrix *Γ*_*α*_. Therefore, it is rather simple to adjust the conductance and the thermoelectric quantities in such a complex system. Under the condition of *μ*=0, the antiresonance state locate only at positive *ε*_0_ side. Figure 5a and b shows the Fano antiresonance valley in the conductance. The inset in Fig. 5a shows the Fano line-shape of the conductance in a large dot level regime. Unlike the case of *t*_0_=0 in which the zero point of the thermopower locates at *ε*_0_=0, that of *t*_0_≠0 is at the antiresonant state, respective to which the thermopower is antisymmetric. For the case of *p*=0, the zero points of the thermopowers of both spin component are at *ε*_0_=0.09 as shown in Fig. 5c and d. With increasing *p*, they are separated and shifted to opposite directions of 0.09. A broad peak with positive and negative values emerge at the two sides of the zero points, respectively. It is worth mentioning that the value of the thermopower is neglectable small in the other dot level regimes, which is shown in the inset of Fig. 5c. The shifting of the zero points as well as the peaks in the thermopowers brings about two interesting results. One is the 100 *%* spin-polarized thermopower when the peaks of *S*_*↑*_ and *S*_*↓*_ are fully separated in energy space by rather large *p* value. See for example the blue dash-dotted line in Fig. 5c and d for *p*=0.4. At the right side of *ε*_0_=0.09, the value of *S*_*↓*_ approaches to zero but *S*_*↑*_ has two sharp peaks. Whereas at the left side of *ε*_0_=0.09, the spin-down thermopower *S*_*↓*_ has two peaks with almost zero *S*_*↑*_.

**Fig. 6:**
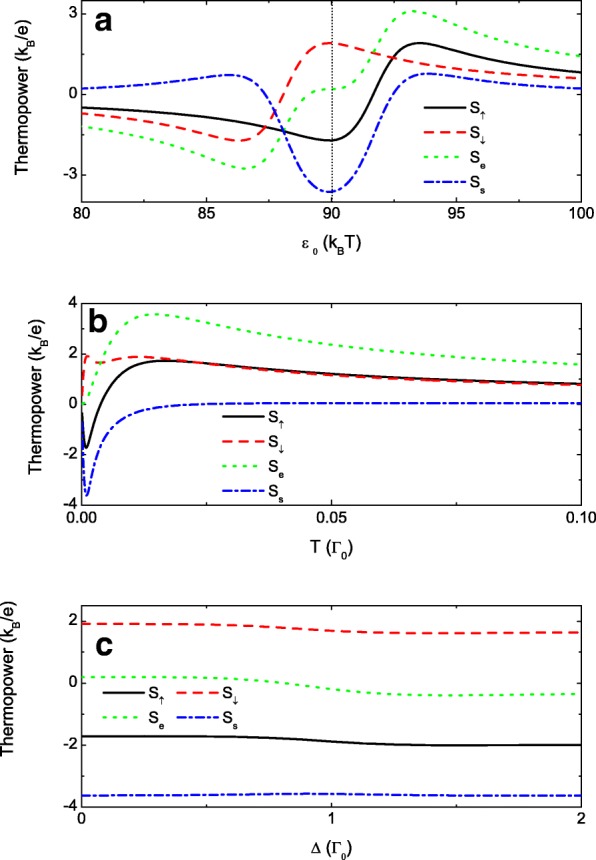
Quantum regulations of the thermopowers. The thermopowers varying with the dot level in **a**, the temperature in **b** and the level detuning in **c**. Other parameters are *p*=0.02, *t*_0_=1, and *t*_*c*_=0.3. The dot level in **b**and **c** is chosen as *ε*_0_=0.09*Γ*_0_. The level detuning *Δ*=0 in **a** and **b**, and the temperature is *T*=0.001 in **a** and **c**

The other interesting result is the pure spin thermopower, i.e., *S*_*s*_=*S*_*↑*_−*S*_*↓*_≠0 while *S*_*e*_=*S*_*↑*_+*S*_*↓*_=0, or pure spin current in closed circuit under finite thermal bias [58]. It means that the spin-up and spin-down thermopowers with equal magnitude are opposite in signs. The magnitude of *S*_*s*_ is maximized when the sharp peaks in the spin-down and spin-up thermopowers with opposite signs meet at the same *ε*_0_ by adjusting the spin-polarization of the interdot couplings *p*. As shown in Fig. 6a, the zero points as well as the peaks in *S*_*↑*_ and *S*_*↓*_ are respectively shifted to the right and left sides of *ε*_0_=90*k*_*B*_*T* due to *p*≠0. As a result of it, the negative peak in the spin-up thermopower and the positive peak in the spin-down one emerge simultaneously around *ε*_0_=90*k*_*B*_*T* inducing the pure spin thermopower. This usually occurs for small *p* because the two narrow peaks in *S*_*σ*_ are very close to the zero points, which is confirmed by the blue dash-dotted line in Fig. 6a with *p*=0.02. To clearly show the small energy dominant, we choose *k*_*B*_*T* as the energy unit in it. We emphasize that this pure spin thermopower may be obtained with very small spin-polarization of the interdot coupling which is realizable by applying a weak magnetic field on the tunnel barriers. Moreover, the magnitude of the pure spin thermopower is as large as the charge one (the green dotted line).

Finally, we present the spin-resolved, pure spin and the charge thermopowers varying with the temperature *T* and the level detuning *Δ* in Fig. 6b and d, respectively. The dot level *ε*_0_ is chosen as 0.09 to focus on the Fano antiresonance valley. Figure 6b shows that at low temperature *S*_*↑*_ and *S*_*↓*_ develop peaks with opposite signs denoted by the solid and dashed lines, resulting in quite large pure spin thermopower *S*_*s*_ (blue dash-dotted line). Now the charge thermopower *S*_*e*_ can be very small as shown by the green dotted line. With increasing temperature, the Fano effect is destructed by the carriers’ random thermal motion, and the peaks in *S*_*σ*_ are smeared out. As a result of it, the difference between *S*_*↑*_ and *S*_*↓*_ is undistinguishable, and the pure spin thermopower approaches to zero. Figure 6d shows that the pure spin thermopower is robust against the difference between the dot levels *Δ*. This is consistent with the result from Eq. (7) that the Fano antiresonant state is independent of dots 1 and 2.

## Conclusions

In conclusion, we have studied the properties of the electric conductance and the thermopower in a TQDs connected either serially or circularly with spin-dependent interdot couplings. Particular attention is paid on the generation of 100 *%* spin-polarized and pure spin thermopowers. It is found that the former can be realized in the serial TQDs configuration with sufficiently large interdot coupling spin polarization when the dots are rather strongly coupled to each other. Whereas if the dots are weakly coupled, giant 100 *%* spin-polarized thermopower can be realized under very small interdot coupling spin polarization. When the dots are in circular configuration, the thermopower is antisymmetric with respective to the Fano antiresonance state around which the thermopower develop sharp peaks. By changing the spin-polarization of the interdot couplings, the peaks in spin-up and spin-down thermopowers are shifted to opposite directions in the QDs levels regime. Now the 100 *%* spin-polarized and pure spin thermopowers can be realized in a quite easy way. The present results can be obtained under small value of the spin polarization of the interdot couplings, which is favorable in experiments.
